# Experiments in Cholera

**Published:** 1856-08

**Authors:** 


### CHOLERA TRANSMITTED FROM MAN TO THE FOWL, BY MEANS OF CHOLERÆIC DISCHARGES, MIXED WITH FOOD:

BY DR. CHARCELLAY.

Being Experiments made at the Ambulance of St. Etienne, by MM. Resnoneau and Homo, Heads of the Clinic, and Students of l'Hospital General of Tours. Read before the Societe Medicale of the Department of Indre et Loire. Translated from the Gazette Hebdomadaire, April, 1856: by M. Morton Dowler, M. D., New Orleans.

On the 23d of August, 1854, the ambulance of the general Hospital of Tours, having become insufficient for the number of Cholera patients furnished by the city and by the hospital inmates, the city government, co-operating with the administrative commission of this establishment, decided on the erection of a supplementary ambulance for the exclusive reception of patients from the city and suburbs. This addition to the establishment was immediately erected in the public school-house of St. Etienne, on Grammont street. In a little inner court were placed three pigeons, and three domestic fowls, one cock and two hens.

For the space of thirteen days, patients were received and treated at the ambulance of St. Etienne, without any of the fowls presenting the least symptom of disease, though there had been given them, at two different times, a paste of crumbs of bread mixed with the alvine dejections furnished by the cholera patients, or found in the intestines of dead subjects. It is true that these animals, and especially the pigeons, evinced a repugnance to this aliment. The cock and the two hens eat of it many times, and did not appear to suffer any inconvenience. The experiment appeared to promise no result, when, on the 14th day, at midnight, one of the hens showed symptoms of illness.

She seemed sad, depressed, caring little to seek her food, being indifferent to what was passing around her. Her gait was slow and tottering, her wings slightly drooping were no longer in contact with the body; her feathers, chiefly those of the back and neck, had changed from their inclined position, and stood erect, on the surface of the body. Toward seven o'clock in the evening the hen retired to her nest, where, at nine o'clock, she had neither mounted on her perch, nor lain down on the ground; but was standing up motionless, neither seeking to avoid or fly the hand that seized her.

Then were witnessed symptoms truly cholerӕic, which supervened a few minutes before death. Thus the hen, which was cold, was crouched down on her belly supporting her beak, and even her head, on the ground. She turned herself on her side, showing convulsive movements in her feet, thighs, and wings, fluttered and twisted herself, and ejected many times, by vomiting efforts, a gluey, viscous, whitish, and lightly frothy fluid. She had also numerous fluid, alvine dejections, of a yellowish white, and very fœtid, she raised herself and was violently agitated on her feet, as if she had cramps. The heat of the body was notably diminished. The crest, from being of a lively red, and even of a deep violet, wrinkled, flaccid and falling, put on a bluish tint, more and more marked. The skin itself became cyanosed, and the feathers stood erect. At last the hen showed the final convulsions, and died, elongating and turning the neck. This little scene continued for two or three minutes.

*Autopsy*, made fifteen hours after death, September 7, at 12 o'clock. at an extremely high temperature. The crest, cyanosed and pale, was of a more violaceous color than the eyelids and commissures of the beak. The skin had retained the blueish tint that it had first assumed, and the flesh also presented this color, though less marked. The margin of the beak and all the buccal cavity were moistened with a stringy citron colored fluid. The epithelium of the point of the tongue was white, and remarkably indurated. The feathers around the anus, were soiled by the dried matter of the dejections—matter much resembling a deposit of plaster, lightly colored yellow. No spots or ecchymoses whatever were found on the body.

An incision made into the abdominal cavity, gave exit to a little albuminous, yellow fluid, of an offensive odor—of some consistence, and bedewing the greater part of the abdominal organs. The anterior face of the liver was covered with a thick coating of this fluid, lightly adhering to its tissue. As to the liver itself, the character of its color and structure seemed in no way modified. Nevertheless, it was friable, and little consistent. We found nothing which appeared abnormal in the spleen, the kidneys or the pancreas. In the oviduct there was a manifestly injected condition; but the cause of which was doubtless owing to the super-excitement of the organ at this epoch, which was with the animal that of regular daily laying eggs. The opening of the crop, the gizzard, and the ventriculus succenturiatus, offered nothing special. It was otherwise, however, with the small intestine, which, through the extent of about from 20 to 25 centimetres, (7 plus to 8 plus inches,) beginning at the gizzard, presented four large acchymosed spots, very distinct on the peritoneal, but much more so on the mucous surface, on which, however, we did not find the little sanguineous granulations, often observed in the intestines of gallinaceous animals, which died during the epezootic of 1851. While the isolated follicles of Brunner were numerously enough affected to constitute an evident psorentery, some of the *plaques* of Peyer appearing lightly raised, without there being evidence sufficient to indicate a real morbid condition. The matter contained in the intestine, and the cæci were in every respect similar to those which had been expelled *per annum* during life. The heart and lungs, which were exposed with care, did not present any ecchymosis or other morbid alteration; but the blood contained in the right ventricle was found in the form of black and resistent coagula, the left ventricle being empty. We did not inquire into the degree of adhesion of the blood with the internal coat of the veins; nor into the influence that the prolonged presence of the blood in these vessels might have had on the color of the membranes, composing the tissue of the venous parietes.

In the nervous system we saw nothing which appeared worthy of remark.

At very nearly the same period, that is to say, during the first days of September, the other hen and the cock, as also two or three of the pigeons, manifested a severe thirst, showing at the same time diminished appetite. During the 8th, 9th, and 10th of the month, the cock had completely lost his voice, and made ineffectual efforts to crow.

The following is an extract from the daily notes made at first by M. Homo, before the occurrence of the grave premonitory symptoms of cholera, which forced him to abandon the ambulance of St. Etienne, and to abruptly break off a service which was supplied by M. Paumier, first substitute student, assisted by M. Carrignon.*

* In consequence of their courageous devotedness, these two students have experienced severe and repeated premonitory symptoms of the epidemic scourge. Some months later, M. Paumier was attacked with typhoid fever; as also were many persons whose service called them to take up an abode, more or less prolonged, amidst the cholerogenic foyers.

Monday, Sept. 4.—The white hen and cock have refused to take the paste; whilst the black hen has eaten of it at once, and has rejected it immediately.

Tuesday, Sept. 5.—The black hen has taken of it three times, and then leaves it without returning to the plate. The cock has partaken of it freely, five or six times; the white hen refuses to eat of it.

Wednesday, Sept. 6.—The female nurse at noon perceived that the black hen was sick; though the woman, did not mention it till six o'clock in the evening. This hen was then crouched on the straw, in the roost, where she was accustomed to retire in the night. She frequently opened her beak, and appeared to respire with great difficulty.

These different experiments, which were continued for three weeks, in August and September, were conducted with the greatest precaution; and ought to afford all possible guarantees as to authenticity. The hens did not go out of the yard, and they were fed in the presence of the nurses or students. The three pigeons experimented on, were retained in a cage; and it was so arranged that it was impossible for the other pigeons to have access to the cholaræic paste.

All I have here detailed relates only to the introduction of choleræic matter into digestive organs. Another order of experiments projected by me towards the close of these, related to the subject of the inoculation with choleræic fluid; as dejections, blood, etc. But circumstances existed which prevented me from putting my design into execution. Thus, aside from the grand reasons before related—the engrossing duties—the fearful agitation of the masses during epidemic cholera, it was quite necessary that I should have an isolated location, at a certain distance from the city, in order to withdraw from the epidemic influence, the animals to be experimented upon. At these calamitous periods, difficulties such as these constitute obstacles truly insurmountable to the provincial practitioner, whose time is entirely employed in frequent visits to the hospital, or other public establishment, by private practice, or municipal service to cholera patients at their domicles. Moreover, it became impossible to proceed with these experiments, as most happily our epidemic is at an end.

